# Contributions of Glucose and Hemoglobin A_1c_ Measurements in Diabetes Screening

**DOI:** 10.1093/ajcp/aqab106

**Published:** 2021-08-31

**Authors:** Lee H Hilborne, Caixia Bi, Jeff Radcliff, Martin H Kroll, Harvey W Kaufman

**Affiliations:** 1 Department of Pathology and Laboratory Medicine, University of California, Los Angeles, CA, USA; 2 Quest Diagnostics, Secaucus, NJ, USA

**Keywords:** Glucose, Hemoglobin A_1c_, HbA_1c_, Diabetes, Screening

## Abstract

**Objectives:**

Given the long-term consequences of untreated diabetes, patients benefit from timely diagnoses. Payer policies often recognize glucose but not hemoglobin A_1c_ (HbA_1c_) for diabetes screening. This study evaluates the different information that glucose and HbA_1c_ provide for diabetes screening.

**Methods:**

We conducted a retrospective review of national clinical laboratory testing during 2020 when glucose and HbA_1c_ were ordered for routine diabetes screening, excluding patients with known diabetes, out-of-range glucose, or metabolic syndrome.

**Results:**

Of 15.47 million glucose and HbA_1c_ tests ordered simultaneously, 672,467 (4.35%) met screening inclusion criteria; 116,585 (17.3%) were excluded because of diabetes-related conditions or the specimen was nonfasting, leaving 555,882 result pairs. More than 1 in 4 patients 60 years of age or older with glucose within range had an elevated HbA_1c_ level. HbA_1c_ claims were denied more often for Medicare beneficiaries (38,918/65,273 [59.6%]) than for other health plans combined (23,234/291,764 [8.0%]).

**Conclusions:**

Although many health plans do not cover HbA_1c_ testing for diabetes screening, more than 1 in 4 glucose screening patients 60 years of age or older with an in-range glucose result had a concurrent elevated HbA_1c_ result. Guideline developers and health plans should explicitly recognize that glucose and HbA_1c_ provide complementary information and together offer improved clinical utility for diabetes screening.

**Key Points**

- Glucose and hemoglobin A_1c_ (HbA_1c_) are frequently discrepant when screening patients for diabetes, particularly in patients 60 years of age and older.
- Depending solely on glucose or HbA_1c_ may fail to recognize at-risk patients who could benefit from early intervention.
- Claims denials are more common for Medicare beneficiaries than for patients with other health plan coverage.

## Introduction

Diabetes is the seventh-leading cause of death in the United States, accounting for more than 3% of all deaths in 2017.^1^ Diabetes also contributes to heart, cerebrovascular, and kidney diseases. Diabetes disproportionately affects older people (≥50 years of age) and people of color.^2^ Optimal diabetes screening facilitates early intervention to mitigate progression of prediabetes and reduces the long-term consequences of diabetes.^3^

Diagnostic criteria for diabetes have been refined over the years.^4^ Initial criteria were primarily based on measuring glucose, but point glucose measurement is problematic because some patients with impaired glucose tolerance or diabetes have glucose levels within the reference range at the time of the measurement. Detection of impaired glucose metabolism, therefore, benefits from a diagnostic approach that simultaneously measures long-term glucose exposure.

In 1969, Rahbar described an increase in an “unusual” hemoglobin in patients with diabetes, now recognized as glycated hemoglobin, or hemoglobin A_1c_ (HbA_1c_).^5^ HbA_1c_ reflects average blood glucose over several months, whereas glucose measurement represents a specific point in time. HbA_1c_ measurement has become standard practice for the evaluation of diabetes control in patients with known diabetes.^6^ HbA_1c_ measurement is also useful for diabetes screening.^7^ In 1993, the American Medical Association’s *Current Procedural Terminology* Editorial Panel granted a category I code for reporting HbA_1c_. Glucose and HbA_1c_ continue to be reimbursed, with appropriate indications and intervals, by Medicare and other insurers.^8,9^

Glucose measurement is approved by Medicare as a screening benefit for at-risk asymptomatic patients without diabetes when reported with *International Classification of Diseases, Tenth Revision (ICD-10)* code Z13.1, “Encounter for screening for diabetes mellitus.” ^10^ HbA_1c_ measurement, however, is presently not covered for screening (Z13.1).^9^ Medicare applies specific criteria to determine whether a screening service, generally an uncovered benefit, will be covered. Specifically, the service must be (1) reasonable and necessary for the prevention or early detection of illness or disability, (2) recommended with a grade of A or B by the United States Preventive Services Task Force (USPSTF), and (3) appropriate for Medicare beneficiaries.^11^

Asymptomatic individuals who present for routine screening generally receive diagnostic laboratory tests that include a basic or comprehensive metabolic panel in addition to other medically appropriate services, such as HbA_1c_ testing. USPSTF guidelines indicate that screening for glucose abnormalities may include either glucose or HbA_1c_ (grade B).^12^ The “*or”* implies that HbA_1c_ may be duplicative when accompanied by a concurrent glucose measurement. Glucose is a component of commonly ordered metabolic panels.

On March 16, 2021, the USPSTF released a revised draft of *Screening for Prediabetes and Type 2 Diabetes Mellitus.*^13^ The proposed revisions continue to recommend diabetes screening for at risk-patients and states that moderate net benefit exists when screening is coupled with effective preventive measures. The revised recommendation states that screening is effective in younger at-risk populations (adults aged 35 to 70 years who are overweight or obese). As proposed, the recommendations acknowledge the benefits of HbA_1c_ screening and discuss the diagnosis of prediabetes or diabetes using a fasting glucose, HbA_1c_, or oral glucose tolerance test. The recommendations do not, however, address situations where glucose and HbA_1c_ results are discrepant with respect to disease classification when used for asymptomatic population screening.

To assess the potential impact of excluding HbA_1c_ as a screening benefit, we evaluated the frequency of discrepant glucose and HbA_1c_ results in patients screened for diabetes.

## Materials and Methods

Deidentified glucose and HbA_1c_ results from individuals tested at 1 of 10 geographically distributed regional laboratories of a national reference laboratory during calendar year 2020 were extracted for analysis. Results were included if glucose and HbA_1c_ tests were ordered simultaneously. Paired results with the *ICD-10* diabetes screening diagnosis (Z13.1) were included, except when the test requisition also included an existing glucose abnormality (diabetes [E08-E11], abnormal glucose [R73], or metabolic syndrome [E88.81]) or the specimen was specifically identified as nonfasting. Data were analyzed by age range and sex. Results were considered discrepant if (1) glucose was within the reference interval (<100 mg/dL, 5.55 mmol/L) but HbA_1c_ indicated prediabetes (5.7%-6.4% [39-46 mmol/mol]) or diabetes (>6.4% [46 mmol/mol]) or (2) glucose was in the prediabetes (100-125 mg/dL [5.55-6.89 mmol/L]) or diabetes (>125 mg/dL [>6.89 mmol/L]) range but HbA_1c_ was within the reference interval.^6^

Claims data were used to explore HbA_1c_ denial rates by payer type. Denials for HbA_1c_ testing were examined when submitted with *ICD-10* diagnosis code Z13.1 and with the exclusions previously noted. Claims were categorized as Medicare, Medicare Advantage, Medicaid, Managed Medicaid, or commercial insurance. Self-pay and client-billed claims were excluded because they are not adjudicated against payer policies. Also excluded were claims that were not fully adjudicated at the time of the study (eg, in appeal or pending) or denied for reasons other than diagnosis codes submitted.

This study was deemed exempt by the Western Institutional Review Board (Puyallup, WA).

## Results

Glucose and HbA_1c_ tests were ordered together 15,468,174 times during the study period; 26,043 (0.16%) result pairs were excluded for missing demographic or payer data. Paired results that included *ICD-10* code Z13.1 totaled 672,467 (4.4%). Of these, 74,334 (11.1%) were excluded because of a reported *ICD-10* code suggesting a glucose abnormality and 42,251 (6.2%) were identified as nonfasting. Of the remaining 555,882 result pairs (227,072 [40.8%] from females), 407,967 (73.4%) had glucose within the reference range, of which 61,042 (15.0%) had elevated HbA_1c_. Conversely, of 147,915 (26.6%) pairs with elevated glucose, 71,991 (48.7%) had HbA_1c_ levels within the reference interval **Table 1**.

**Table 1:** Discrepant Glucose and HbA_1c_ Results (Calendar Year 2020) When Submitted With Diabetes Screening *ICD-10* Code Z13.1 by Payer Type, Excluding Patients With a Diabetes-Related Condition

Age Group, y	Patients, No.	Glucose <100 mg/dL (5.55 mmol/L)		Glucose ≥100 mg/dL (5.55 mmol/L)	
		Patients (%)	HbA_1c_*>*5.7% (39 mmol/mol), % of Patients (95% CI)	Patients, No. (%)	HbA_1c_ <5.7% (39 mmol/mol), % of Patients (95% CI)
All ages	555,882	407,967 (73.4)	15.0 (14.9-15.0)	147,915 (26.6)	48.7 (48.5-48.8)
<10	1,868	1,719 (92.0)	3.7 (3.4-4.2)	149 (8.0)	92.6 (90.5-94.8)
10-20	21,605	19,368 (89.6)	4.7 (4.6-4.9)	2,237 (10.3)	79.6 (78.8-80.5)
20-29	62,846	55,861 (88.9)	4.4 (4.3-4.5)	6,985 (11.1)	73.1 (72.6-73.7)
30-39	96,315	79,327 (82.4)	8.2 (8.1-8.3)	16,988 (17.6)	62.6 (62.3-63.0)
40-49	116,096	86,973 (74.9)	13.9 (13.8-14.0)	29,123 (25.1)	52.2 (51.9-52.5)
50-59	123,465	82,359 (66.7)	21.6 (21.4-21.7)	41,106 (33.3)	43.9 (43.6-44.1)
60-69	89,028	55,227 (62.0)	24.7 (24.5-24.9)	33,801 (38.0)	42.0 (41.8-42.3)
70-79	32,935	20,040 (60.8)	27.5 (27.1-27.8)	12,895 (39.2)	39.5 (39.0-39.9)
>80	11,724	7,093 (60.5)	29.8 (29.2-30.3)	4,631 (39.5)	38.5 (37.8-39.2)

HbA_1c_, hemoglobin A_1c_; ICD-10, International Classification of Diseases, Tenth Revision.

**Figure 1:**
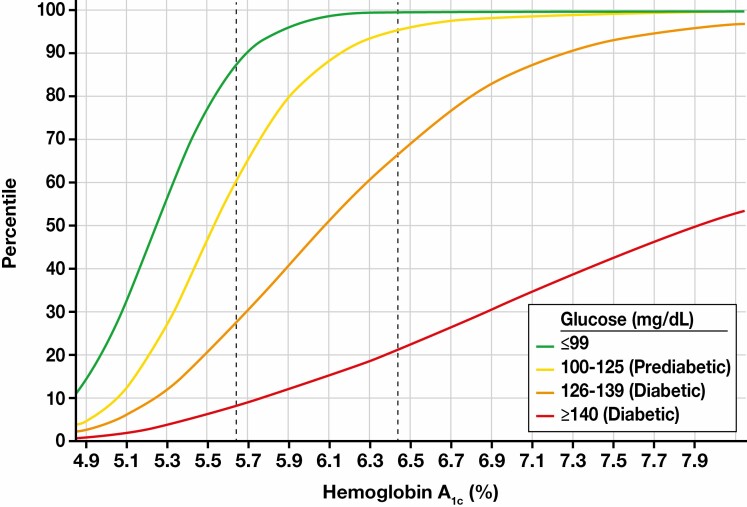
Cumulative distribution of hemoglobin A_1c_ (HbA_1c_) based on glucose categories (within reference interval, prediabetes, diabetes) from all 555,882 paired HbA_1c_ and glucose results. Vertical dashed lines highlight diagnostic HbA_1c_ thresholds for prediabetes (left) and diabetes (right), calendar year 2020.

Having an in-range glucose level was more common in women (255,760/328,810 [78%]) than in men (152,207/227,072 [67%]). The frequency of elevated HbA_1c_ (≥5.7% [39 mmol/mol]) among pairs with in-range glucose was slightly higher for women (15.9% [95% CI, 15.8%-16.0%]) than for men (14.4% [95% CI, 14.4%-14.5%]). HbA_1c_ levels increased with increasing age group **Table 1 **; among patients 60 years of age and older with in-range glucose, 25.8% (21,266/82,360) had elevated HbA_1c_ levels. Although most (59,041 [96.7%]) patients with a glucose level under 100 mg/dL (5.55 mmol/L) and an elevated HbA_1c_ level had HbA_1c_ levels in the prediabetes range (5.7%-6.0% [39-42 mmol/mol]), some (2001 [3.3%]) had HbA_1c_ levels in the overt diabetes range (>6.5%) (**Figure 1 **, Supplementary Table 1, Supplementary Figure; all supplemental material can be found at *American Journal of Clinical Pathology* online).

Few differences existed by payer type except for Medicare: 21.7% of Medicare beneficiaries and 14.7% of patients with other insurance had glucose levels within the reference range but elevated HbA_1c_ levels that could suggest either prediabetes or diabetes. Coverage denial rates for HbA_1c_ were substantially higher for Medicare fee-for-service (FFS) (21,045/31,734 [66.3%]) and Medicare Advantage (17,873/33,539 [53.3%]) beneficiaries than for Medicaid (1307/18,901 [6.9%]), Managed Medicaid (7872/33,832 [23.3%]), or commercial insurance (14,055/239,031 [5.9%]) patients. (Supplementary Table 2).

## Discussion

This study identified discrepancies between glucose and HbA_1c_ levels to identify patients at risk for prediabetes or diabetes. The USPSTF last issued a grade B recommendation favoring “screening for abnormal blood glucose as part of cardiovascular risk assessment in overweight adults aged 40 to 70, followed by intensive behavioral counseling to promote a healthful diet and physical activity when glucose abnormalities are found.” ^12^ In 2021, the USPSTF proposed lowering the recommended screening age to begin diabetes screening at 35 years.^13^ Regarding screening, the USPSTF notes that “glucose abnormalities can be detected by measuring HbA_1c_ or fasting plasma glucose or with an oral glucose tolerance test.” ^13^

Our findings suggest that glucose or HbA_1c_ testing alone when screening for abnormalities of glucose metabolism could miss or delay diagnosis of prediabetes or diabetes for many patients. Medicare coverage of glucose^8^ but not HbA_1c_^9^ testing would be sufficient if glucose and HbA_1c_ were clinically equivalent for screening. Our data suggest otherwise. More than 1 in 4 patients 60 years of age and older with glucose within the reference range had elevated HbA_1c_ results. Even among younger patients, 12.9% (54,493/422,195) of individuals who had glucose within the reference range had elevated HbA_1c_ results **Table 1** . These findings align with those of the Canadian Task Force on Preventive Care.^7^

The likelihood of claim denial was approximately 9 times greater for Medicare Advantage beneficiaries than for commercial insurance beneficiaries. Denials were higher for patients with Managed Medicaid than for patients with Medicaid FFS, likely reflecting alignment with payment policies incorporated into Medicaid contracts managed by the same commercial plans that manage Medicare Advantage programs.

Study limitations include lack of clinical data and evaluating only requisitions with *ICD-10* code Z13.1. Although code Z13.1 should, by coding guidelines, exclude patients with known altered glucose metabolism, 11.1% of these requisitions contained a glucose abnormality diagnostic code. The combination of elevated glucose with normal HbA_1c_ levels likely reflects inadequate fasting, transient hyperglycemia, or early-stage disease. Many factors limit the correlation between HbA_1c_ and glycemia (eg, age, ethnicity, hemoglobinopathy), but screening services for patients at high risk for important conditions should focus on sensitivity over specificity, with further investigation or follow-up based on clinical considerations.

Our findings suggest that glucose and HbA_1c_ are complementary when screening for glucose abnormalities, and excluding either test could delay diagnosis and management in many patients. Further studies should assess the time delay caused by routinely excluding HbA_1c_ as part of diabetes screening and the magnitude of the differences. We encourage guideline developers and health plans to recognize that glucose and HbA_1c_ provide complementary information and together offer improved clinical utility for diabetes screening.

## Supplementary Material

aqab106_suppl_Supplementary_Material
