# Supplementary material for: Contributions of Glucose and Hemoglobin A1c Measurements in Diabetes Screening
Source: Am J Clin Pathol. 2021 Aug 31;157(1):1–4. doi: 10.1093/ajcp/aqab106 (PMC8742979; doi:10.1093/ajcp/aqab106)

Supplementary Table 1. Summary of results by diabetes classification category (numbers in parentheses represent column percent)

Supplementary Table 2 – Claims paid and denial rate for HbA_1c_ (January-December 2020) when submitted with diabetes screening ICD-10 code Z13.1 by payer type, excluding patients with a diabetes related condition

| **Payer Class** | **Total Claims** | **Claims Paid** | **Claims Denied** | **Denial Rate** |
| --- | --- | --- | --- | --- |
| **Medicare-Fee for Service** | 31,734 | 10,689 | 21,045 | 66.3% |
| **Medicare Advantage** | 33,539 | 15,666 | 17,873 | 53.3% |
| **Medicaid** | 18,901 | 17,594 | 1,307 | 6.9% |
| **Medicaid-Managed** | 33,832 | 25,960 | 7,872 | 23.3% |
| **Commercial Insurance** | 239,031 | 224,976 | 14,055 | 5.9% |

Supplementary Figure. Scatter density plot using a random selection of 20,000 observations from all 555,882 paired HbA_1c_ and glucose specimens, year 2020


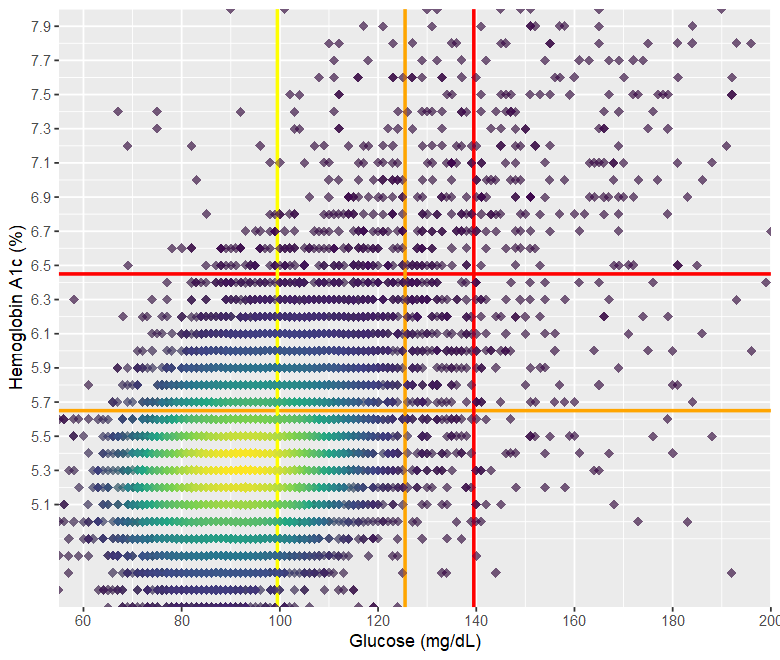

Supplement: aqab106_suppl_Supplementary_Material [file aqab106_suppl_supplementary_material.docx]
